# Perforated Meckel's diverticulum presenting with combined bowel and urinary obstruction and mimicking Crohn's disease: a case report

**DOI:** 10.1186/1752-1947-4-264

**Published:** 2010-08-11

**Authors:** Banny S Wong, David W Larson, Thomas C Smyrk, Amy S Oxentenko

**Affiliations:** 1 Division of Gastroenterology and Hepatology, Department of Internal Medicine, Mayo Clinic, 200 First Street SW, Rochester, Minnesota 55905, USA; 2 Division of Colon and Rectal Surgery, Department of Surgery, Mayo Clinic, 200 First Street SW, Rochester, Minnesota 55905, USA; 3 Department of Anatomic Pathology, Mayo Clinic, 200 First Street SW, Rochester, Minnesota 55905, USA

## Abstract

**Introduction:**

Meckel's diverticulum is a common congenital anomaly of the gastrointestinal tract, but is an uncommon cause of serious complications in adults. Although cases of patients with hemorrhage, bowel obstruction or perforation associated with Meckel's diverticulum have been reported, there have been no prior reports of patients with combined urinary and bowel obstruction due to abscess formation.

**Case presentation:**

We describe the case of a 21-year-old man with a history of recurrent papillary thyroid cancer, but no prior abdominal surgeries, who presented with a one-month history of rectal pain and new-onset obstipation with urinary retention. He reported night sweats and weight loss, and had a second-degree relative with known Crohn's disease. A digital rectal examination was notable and revealed marked tenderness with proximal induration. A computed tomography scan of the patient's abdomen revealed a large, complex, circumferential perirectal abscess compressing the rectal lumen and base of the urinary bladder, associated with terminal ileal thickening and an ileocecal fistula. A flexible sigmoidoscopy with an endorectal ultrasound scan displayed a complex abscess with extensive mucosal and surrounding inflammation. An exploratory laparotomy revealed a Meckel's diverticulum with a large perforation at its base, positioned near the ileocecal fistula and immediately superior to the perirectal abscess. The section of small bowel containing the Meckel's diverticulum, the terminal ileum, and the cecum, were all resected, and the abscess was debrided.

**Conclusions:**

Pre-operative diagnosis of Meckel's diverticulum can be difficult. If the nature of the complication makes ultimate surgical management likely, an early laparoscopic or open exploration should be performed to prevent the morbidity and mortality associated with late complications.

## Introduction

Meckel's diverticulum is a congenital anomaly found in approximately 2% of the general population. Complications develop in only 4% of patients with this malformation, with most cases presenting in childhood [1]. Complications of Meckel's diverticulum include hemorrhage, bowel obstruction, inflammation, and perforation. All of these complications can be challenging to diagnose because patients may present with non-specific symptoms, which produce a clinical picture that can mimic other more common gastrointestinal disorders [2]. We report an unusual case of a patient with a perforated Meckel's diverticulum and secondary perirectal abscess formation who presented with rectal pain, obstipation, and urinary retention. Clinical considerations included Crohn's disease and malignancy. A definitive diagnosis and treatment for this patient could not have been achieved without a surgical approach.

## Case presentation

A 21-year-old Caucasian man was transferred to the Clinic for an evaluation of a complex perirectal abscess. The patient had experienced rectal pain with defecation for a month prior to presentation, and was initially treated conservatively for presumed hemorrhoidal disease. His symptoms progressed so that passing flatus alone caused him significant discomfort. He then developed worsening constipation. A rectal examination was notable and revealed marked tenderness and induration. Imaging and a proctoscopic examination under anesthesia were performed at an outside hospital before admission to our clinic, and a complex perirectal fluid was collected but could not be adequately drained. The patient then developed increasing difficulty urinating and a four-day history of obstipation, prompting transfer.

The patient's past medical history was notable for the occurrence of a papillary thyroid carcinoma with cervical lymph node metastases that was diagnosed one year before and treated with a total thyroidectomy and post-operative radioiodine therapy. He had a recent lymph node recurrence prompting a modified radical neck dissection at an outside hospital one week before transfer to our clinic. He had no history of prior abdominal or pelvic surgeries. The only medication he was taking was levothyroxine. He was single, and denied tobacco, alcohol, or drug use. He also denied any rectal instrumentation or anal intercourse. His family history was notable for a paternal uncle with Crohn's disease, and a paternal grandmother with a history of resected thyroid cancer. A review of the patient's systems was positive for a 10 kg weight loss in the past month, anorexia, a decrease in stool caliber, fatigue, and painful urination, in addition to the presenting complaints. He denied having a fever, but did complain of night sweats.

A physical examination revealed a tall, thin man in no acute distress. His maximum body temperature was 37°C, with a blood pressure of 100/60 mmHg and a heart rate of 88 beats per minute, with normal respiration and oxygen saturation. His abdomen was soft, with normal bowel sounds, no distension, and no palpable masses. He had mild tenderness in his right and left lower quadrants as well as his suprapubic region, but no rebound or guarding. A Foley catheter was put into position. A perianal inspection was negative for fistulae, fissures, or external hemorrhoids. A digital rectal examination could not be completed because of significant tenderness.

**Figure 1:**
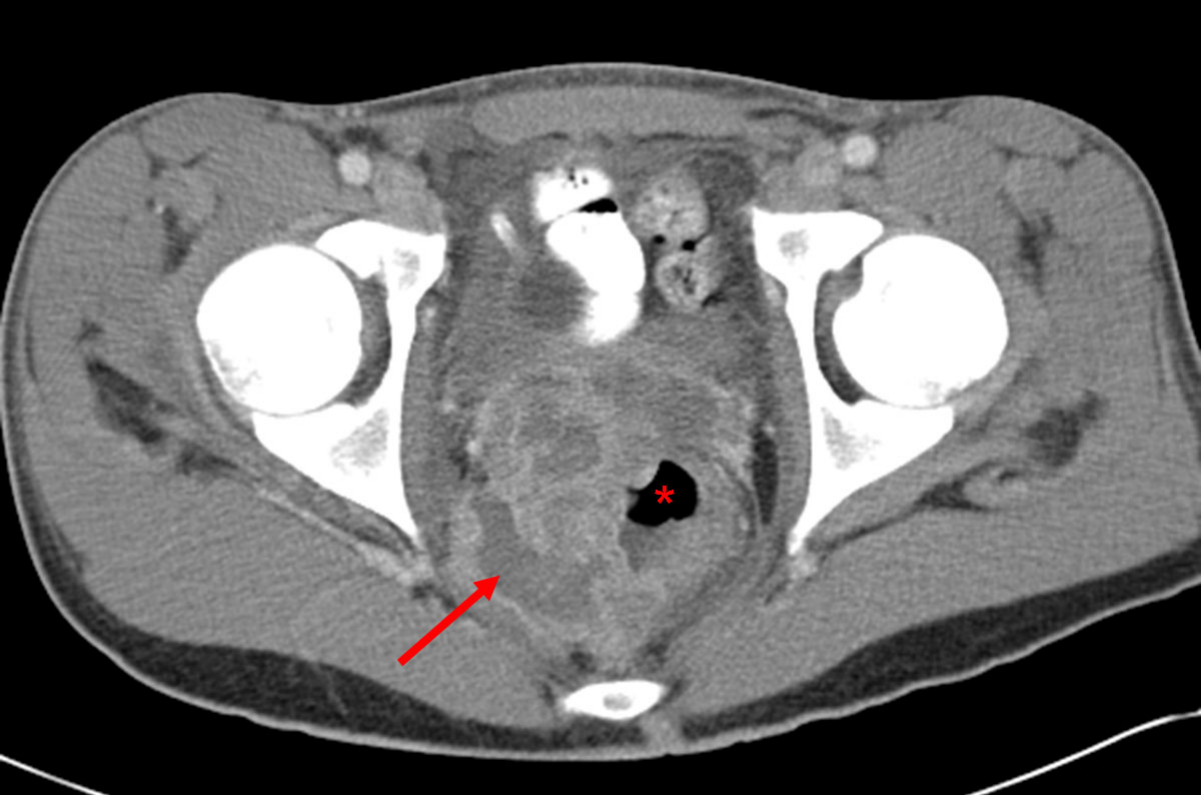
A computed tomography scan of the patient's abdomen and pelvis revealed an extensive, loculated fluid collection (arrow) encircling the distal rectum (*) with surrounding inflammation, consistent with a perirectal abscess. Additionally, a thickening of the wall of the terminal ileum with a fistulous tract from the distal terminal ileum to cecum was noted but is not shown in this figure.

On the day of admission, laboratory tests showed the patient had mild microcytic anemia (hematocrit of 10.5 g/dL and mean cell volume of 79.7 fL) and hypoalbuminemia (3.3 g/dL), with the remainder of the complete blood count, electrolyte level and liver biochemistry being within normal limits. A computed tomography (CT) scan of the patient's abdomen and pelvis demonstrated an extensive, loculated fluid collection encircling the distal rectum, with a large amount of surrounding inflammation. Compression of the patient's distal rectal lumen and bladder neck by the perirectal collection was seen, but adequate bladder decompression was obtained using a Foley catheter. Wall thickening of the terminal ileum and a fistulous tract from the distal ileum to the cecum were noted (Figure 1).

**Figure 2:**
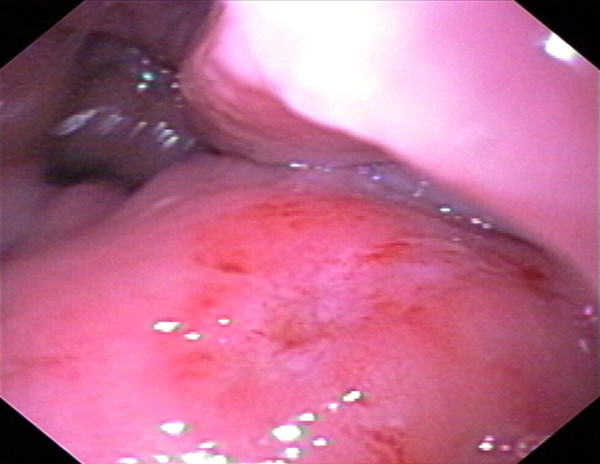
**Endoscopic view of the rectosigmoid mucosa demonstrates erythema and edema, with luminal narrowing due to multiple areas of extrinsic compression from the abscess**.

**Figure 3:**
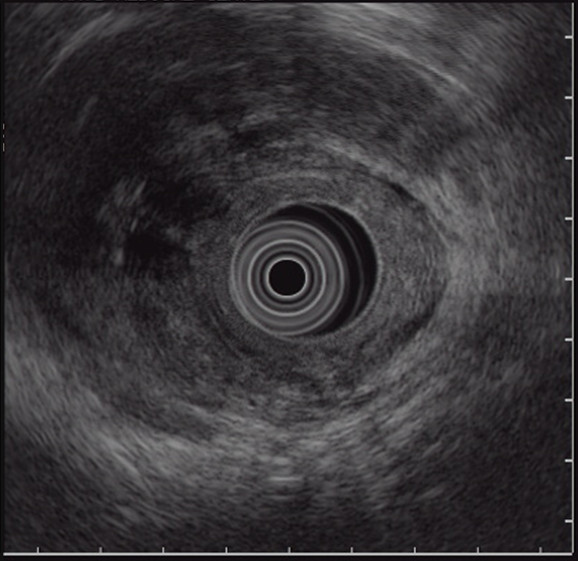
**Endorectal ultrasound image showing a loculated structure, with mobile fluid, solid debris, and significant surrounding inflammation, around the rectosigmoid area**.

A flexible sigmoidoscopy on day two demonstrated an erythematous, edematous rectosigmoid colon with multiple areas of extrinsic compression (Figure 2). Although a colonoscopy was attempted for cecal and ileal inspection and tissue sampling, the patient could not tolerate further advancement of the endoscope. An endorectal ultrasound showed a complex solid and cystic structure surrounding the rectosigmoid area, with mobile fluid, solid debris, and significant surrounding inflammation (Figure 3). The process abutted the sphincteric complex, prostate gland, and bladder. A biopsy of the patient's rectum revealed focal acute inflammation with a poorly formed mucosal granuloma, but no chronic architectural changes. A fine-needle aspirate of the cystic structure produced a yellow, turbid fluid containing many leukocytes and mixed bacterial flora, but no malignant cells.

**Figure 4:**
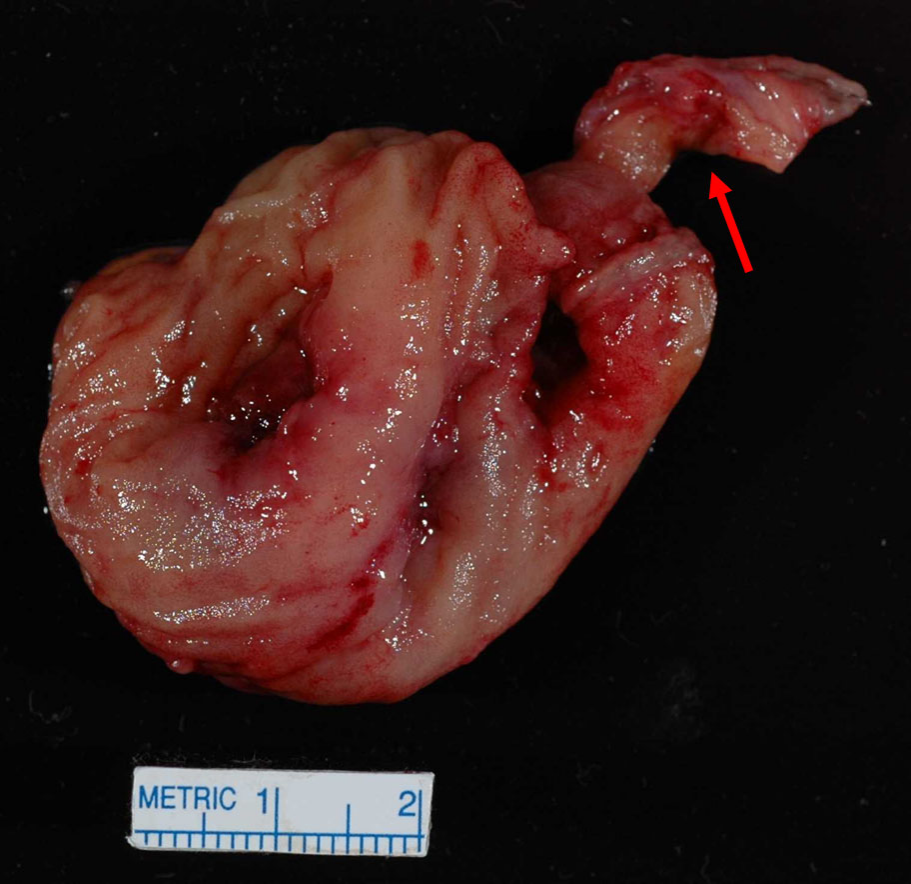
**Gross pathology specimen of the patient's resected bowel reveals a Meckel's diverticulum (arrow) with perforation at its tip, attached to the small intestine**.

On day six of his hospitalization, the patient had an exploratory laparoscopy and the terminal ileum and cecum were found to be densely adhered to the pelvic side wall, with an apparent fistulous tract further fixing the distal ileum to the cecum. Laparoscopic mobilization of the involved structures was not possible, and conversion to laparotomy with a low midline incision was performed. The ileum and cecum were mobilized to expose a large, perirectal and pelvic lateral sidewall abscess, which was thoroughly debrided. A Meckel's diverticulum with a large perforation at its base was densely adherent to an ileocecal fistula, with surrounding inflammation and fibrosis. Given the inability to repair the defect in the terminal ileum from the fistulous opening, resection of a segment of the terminal ileum and cecum was performed with a side-to-side, functional end anastomosis. The portion of small bowel containing the Meckel's diverticulum was also resected, with a primary anastomosis accomplished. On abdominal exploration, no other obvious abnormalities were seen to support a diagnosis of Crohn's disease or malignancy. Fecal diversion was deemed unnecessary because the perforated Meckel's diverticulum was felt to be the underlying source of the patient's symptoms, and resection and debridement with single intraabdominal drain placement had allowed for decompression. Gross pathologic examination of the surgical specimen confirmed a perforated Meckel's diverticulum (Figure 4) with extensive acute inflammation and fibrosis of the adjacent small and large bowels. A histologic examination of the Meckel's diverticulum did not reveal gastric- or pancreatic-type mucosa. There was no histologic evidence to support a diagnosis of Crohn's disease. Specifically, there was no chronic inflammation, crypt architectural distortion or additional granulomas seen in the area surrounding the Meckel's diverticulum, in the ileum or the cecum. In addition, no evidence of malignancy was seen.

Postoperatively, the patient recovered uneventfully. He initially received three days of total parenteral nutrition but was subsequently advanced to a normal diet without difficulty prior to hospital discharge on day 14. He regained normal bowel and bladder function before dismissal. Clinical follow up over the next three years was unremarkable, with no evidence of inflammatory bowel disease or long-term bowel or bladder sequelae.

## Discussion

We report a complicated and unusual case of a patient with a perforated Meckel's diverticulum who presented with obstipation and urinary retention. The patient required an open laparotomy for definitive diagnosis and management.

Complications in patients with Meckel's diverticulum are rare; most patients remain asymptomatic for life [3]. In both adults and children, intestinal obstruction and bleeding have been reported to be two of the most common complications of a Meckel's diverticulum [3-6]. Small bowel obstructions related to Meckel's diverticulum have been reported due to intussusception, incarceration in a hernia sac, or entrapment by an adhesive band, or as being secondary to neoplasm [7]. The pre-operative diagnosis of a patient with Meckel's diverticulum often presents a challenge to the clinician in both children and adults, because presenting symptoms can be non-specific and the differential diagnosis broad [4].

The perforation of a Meckel's diverticulum may mimic acute appendicitis and present as an acute abdomen [6]. In our case, perforation did not produce peritonitis, but presumably led to the formation of an ileocecal fistula and a pelvic abscess via local inflammation, which remained relatively asymptomatic until the abscess became large enough to cause external compression of both the rectum and the bladder neck.

The presence of ectopic gastric mucosa is common in complicated and symptomatic cases of Meckel's diverticulum, including patients with bleeding, inflammation, or perforation [3,4,6]. Interestingly, our patient's Meckel's diverticulum did not contain ectopic gastric or pancreatic mucosa on histologic examination. Other reported etiologies in patients with perforated Meckel's diverticulum include trauma [8], ingested sharp foreign bodies such as a tooth pick [9] or fish bone [3], and tumors such as leiomyosarcoma within the diverticulum [3,10]. In addition, obstruction of the diverticular lumen or diverticular torsion may lead to diverticulitis with inflammation severe enough to lead to perforation, similar to some cases of appendicitis. A Meckel's diverticulitis may conceivably be the source of our patient's perforation given the lack of trauma, foreign body, or neoplasm found on surgical exploration and the histologic examination of the resected specimen.

The initial differential diagnoses for this patient included inflammatory bowel disease (IBD), malignancy, and perforated appendicitis. CT imaging failed to visualize the Meckel's diverticulum, partly because administration of intraluminal rectal contrast was contraindicated with bowel obstruction and a high risk of perforation. A recent study found that CT imaging can be helpful in the diagnosis of patients with Meckel's diverticulitis, but confirmed that bowel obstruction presents a greater diagnostic challenge due to a decreased sensitivity without intraluminal opacification [11]. In our patient, the CT findings of terminal ileal thickening, an ileocecal fistula, and a pelvic abscess increased the suspicion for Crohn's disease.

Endorectal ultrasound findings were more consistent with the appearance of a complicated abscess rather than malignancy. A fine-needle aspirate of the abscess fluid also lacked malignant cytology. Endoscopically, there were no ulcerations or gross findings to support a diagnosis of IBD, and rectal biopsy specimens did not show chronic inflammatory changes. However, difficulty in advancing the colonoscope precluded biopsy of the terminal ileum pre-operatively to rule out Crohn's disease. Ultimately, laparotomy was required both to diagnose and to treat this patient definitively. Surgical pathology showed no evidence of Crohn's disease or malignancy, and the patient continues to do well more than three years post-operatively. Interestingly, reports of patients with Meckel's diverticulum masquerading as Crohn's disease are rare [12], but cases of patients with Meckel's diverticulum associated with confirmed Crohn's disease are not uncommon. However, it is not clear whether the prevalence of Meckel's diverticulum is increased in patients with diagnosed Crohn's disease [13].

In summary, our case illustrates the difficulty in diagnosing a complex case of a patient with a perforated Meckel's diverticulum. Both CT and endorectal ultrasound failed to achieve the diagnosis. Nuclear imaging with a 'Meckel's scan' was not pursued because of the complicated nature of the case. Due to the lack of ectopic gastric mucosa in the resected specimen, the scan would not have assisted in diagnosis even if performed. Laparoscopy did not lead to the diagnosis due to inflammatory adhesions precluding adequate exposure, and therefore, a laparotomy was unavoidable and proved definitive in facilitating both diagnosis and management in our patient.

## Conclusions

As illustrated in our case and supported by other reports, pre-operative diagnosis of patients with Meckel's diverticulum can be challenging. Nuclear imaging using technetium-99 m pertechnetate can be considered for detection of ectopic gastric mucosa associated with many of the complications of Meckel's diverticulum. However, if the nature of the complication is likely to require surgical management, an early laparoscopic or open exploration should be performed to prevent the morbidity and mortality associated with late complications.

## Consent

Written informed consent was obtained from the patient for publication of this case report and any accompanying images. A copy of the written consent is available for review by the Editor-in-Chief of this journal.

## Competing interests

The authors declare that they have no competing interests.

## Authors' contributions

BSW and ASO wrote the manuscript. DWL reviewed the manuscript and provided surgical specimens and expertise. TCS reviewed the manuscript and provided pathology expertise. All authors read and approved the final manuscript.
